# Evaluating pulmonary stenosis and regurgitation impact on cardiac strain and strain rate in a porcine model via magnetic resonance feature tracking

**DOI:** 10.1007/s10554-024-03305-6

**Published:** 2025-01-22

**Authors:** Simon F. Rösel, Sören J. Backhaus, Torben Lange, Alexander Schulz, Johannes T. Kowallick, Kritika Gowda, Julia Treiber, Andreas Rolf, Samuel T. Sossalla, Gerd Hasenfuß, Shelby Kutty, Andreas Schuster

**Affiliations:** 1 University Medical Center Göttingen, Department of Cardiology and Pneumology, Georg-August University, Robert-Koch-Str. 40, 37099 Göttingen, Germany; 2 German Center for Cardiovascular Research (DZHK), Partner Site Göttingen, Göttingen, Germany; 3 Department of Cardiology, Campus Kerckhoff of the Justus-Liebig-University Giessen, Kerckhoff-Clinic, Bad Nauheim, Germany; 4 German Center for Cardiovascular Research (DZHK), Partner Site Rhine-Main, Bad Nauheim, Germany; 5 Department of Medicine, Cardiovascular Division, Beth Israel Deaconess Medical Center and Harvard Medical School, Boston, USA; 6 FORUM Radiology, Rosdorf, Germany; 7 Helen B. Taussig Heart Center, The Johns Hopkins Hospital and School of Medicine, Baltimore, MD USA; 8 Department of Cardiology and Angiology, Medical Clinic I, University Hospital Giessen, Justus-Liebig-University Giessen, Giessen, Germany; 9 FORUM Cardiology, Rosdorf, Germany

## Abstract

**Background:**

Pulmonary stenosis (PS) is common in congenital heart disease and an integral finding in Tetralogy of Fallot (TOF). Pulmonary regurgitation (PR) is more commonly found following surgery in repaired TOF. We aimed to evaluate the haemodynamic effects of PS and PR on cardiac physiology in a porcine model using cardiac magnetic resonance-based feature tracking (CMR-FT) deformation imaging.

**Methods:**

CMR-FT was performed in 14 pigs before and 10–12 weeks after surgery. Surgery included either pulmonary artery banding to simulate PS (*n* = 7), or an incision to the pulmonary valve to simulate PR (*n* = 7). CMR-FT assessment included left and right ventricular global longitudinal (LV/RV GLS) and LV circumferential (GCS) strain and strain rates (SR) as well as left and right atrial reservoir/conduit/booster pump (LA/RA Es, Ee, Ea) strain and SR.

**Results:**

RV GLS was significantly reduced following PS compared to PR induction (PS -7.51 vs. PR -23.84, *p* < 0.001). RV GLS improved after induction of PR (before − 20.50 vs. after − 23.84, *p* = 0.018) as opposed to PS (before − 11.73 vs. after − 7.51, *p* = 0.128). Similarly, RA Es (PS 14.22 vs. PR 27.34, *p* = 0.017) and Ee (PS 8.65 vs. PR 20.51, *p* = 0.004) were decreased in PS compared to PR with detrimental impact of PS (Es before 23.20 vs. after 14.22, *p* = 0.018, Ee before 15.04 vs. after 8.65, *p* = 0.028) but not PR (Es before 31.65 vs. after 27.34, *p* = 0.176, Ee before 20.63 vs. after 20.51, *p* = 0.499).

**Conclusions:**

In a porcine model of RV pressure vs. volume overload, increased after- but not preload shows detrimental impact on RV and RA physiology.

## Introduction

Tetralogy of Fallot (TOF) is the most frequent cyanotic congenital heart defect occurring in about 0.3 per 1000 live births [1]. Pathological alterations include ventricular septal defect, overriding aorta joint to both ventricles, right ventricular outflow tract (RVOT) obstruction with the possible involvement of pulmonary stenosis (PS), and right ventricular (RV) hypertrophy [2, 3]. Treatment of choice for TOF is surgical repair, which should be carried out within the first half year of life with the specific timing depending on clinical presentation [4]. Pulmonary regurgitation (PR) is a noteworthy, potentially serious consequence in repaired TOF [5], as it may carry the risk of RV mechanical and electrical dysfunction mediated by increased volume load [6].

Whilst cardiac load intrinsically regulates cardiac compensation and remodelling, pre- (PR) and afterload (PS) present different haemodynamic challenges and may result in distinctly different patterns [7]. It has been demonstrated that increased afterload primarily leads to concentric hypertrophy, as more force is required to eject blood into the arterial system during systole [5, 7]. In contrast, increased preload heightens the chamber’s wall stress in end-diastole [8] and is associated with eccentric hypertrophy [7]. However, whilst from a haemodynamic viewpoint, concentric hypertrophy allows for a better balancing of wall stress than eccentric hypertrophy, there is evidence to suggest adverse effects for concentric hypertrophy on histological and molecular levels [9].

Cardiac magnetic resonance imaging (CMR) is regarded as the reference standard for assessment and quantification of both ventricular size as well as haemodynamics [10]. Additionally, CMR offers free selection of imaging planes allowing for optimal depiction of complex congenital heart defects [4]. CMR feature tracking (FT) allows deformation assessment of all cardiac chambers [11]. Left ventricular global longitudinal strain (LV GLS) allows a more sensitive detection of ventricular dysfunction than volumetric assessments (e.g., ejection fraction, EF). An underlying reason may relate to subendocardial, longitudinal muscle fiber orientation generating longitudinal strain whilst being a predilection site for early myocardial damage [12]. As such, it was shown that GLS provides additional prognostic information over EF after survived myocardial infarction [12, 13] and in patients with acute heart failure [14]. Atrial strain offers the additional benefit of reflecting (a) diastolic ventricular properties, and (b) atrial reserves to overcome poor ventricular function. Hindered passive ventricular filling is an early finding in diastolic dysfunction quantified by impaired conduit strain [15, 16]. Increased active atrial function may initially compensate for ventricular diastolic dysfunction [17], but deteriorates during further disease progression [16, 18]. Consequently, impaired total strain represents in parts congestion and loss of intrinsic atrial function [16, 19]. As such, strain analyses have been extensively validated and with proven incremental diagnostic and prognostic benefit in numerous diseases such as heart failure with preserved ejection fraction (HFpEF) [12] and ischemic heart disease [13, 20]. Recently, more attention has been given to the study of right cardiac functional quantification. The value of RV and right atrial strains has been not only shown for diseases of the right heart, such as pulmonary hypertension and tricuspid regurgitation, but there is also growing evidence for disease of the entire heart such as heart failure [21–23].

The aim of the present study was to investigate the potentially divergent effects of pressure as opposed to volume overload by detailed cardiac functional quantification in a porcine model of PS compared to PR.

## Methods

### Study population

This was a subanalysis of a previously reported study, extensive details on the study population can be found in the respective publications [24, 25]. Briefly, the present analysis included pre- and post-intervention CMR scans of a total of 14 pigs. All pigs underwent a first CMR scan followed by surgical PS or PR model creation within 24 h. Follow-up CMR was conducted 10–12 weeks later. During the study, pigs’ vital parameters were constantly under observation. The study met approval from the relevant authorities at the University of Nebraska Medical Center and met the requirements laid out in the Guide for the Care and Use of Laboratory Animals.

### PS and PR models creation

The process of PS and PR model creation has been described in detail elsewhere [25]: In brief, for the RV pressure overload model, stenosis of the main pulmonary artery was achieved by surgically employing a band until the intended blood flow velocity was reached. For the RV volume overload model, most of the semilunar leaflets were removed, utilizing a longitudinal surgical access to the RVOT. The respective model’s success was verified echocardiographically.

### Cardiovascular magnetic resonance

The CMR protocol for this study has been described previously [25]: Briefly, CMR studies were performed on a 1.5 Tesla Philips Achieva scanner (Philips Medical Systems, Best, the Netherlands). Cardiac chambers were studied using an electrocardiogram-gated bSSFP sequence during breath hold in 2-chamber (2-CV), 4-chamber (4-CV), and short-axis (SAX) planes, the latter encompassing both ventricles using 8 to 12 parallel slices.

**Fig. 1:**
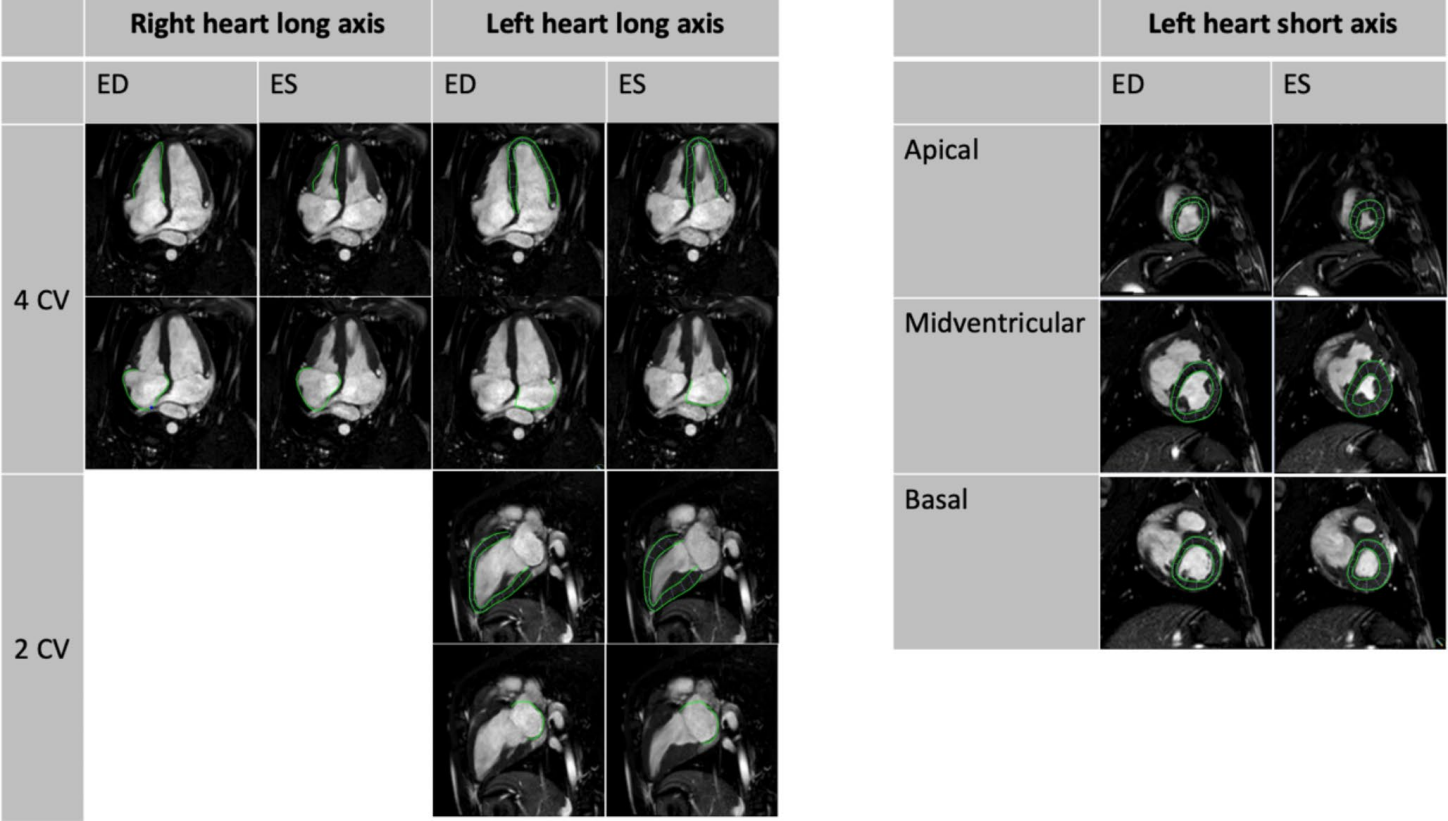
Exemplary endo-/epicardial contours for deformation analysis using CMR-FT Abbreviations: 4 CV: Four chamber view. 2 CV: Two chamber view. ED: Enddiastolic, ES: Endsystolic

CMR-FT was carried out using commercial software (2D CPA MR, Cardiac Performance Analysis, Version 1.1.2; TomTec Imaging Systems, Unterschleissheim, Germany) as previously described [13, 22, 26, 27], Fig. 1. CMR-FT refers to a post-processing technique which allows quantification of strain and strain rates of all cardiac chambers [28]. Strain is used to describe myocardial shortening in longitudinal or circumferential as well as myocardial thickening in radial direction. Beyond total atrial strain, deformation imaging can be used to quantify atrial phasic function. First, reservoir function describes total atrial strain and collection of venous return during ventricular systole (left/right atrial LA/RA Es). Secondly, atrial passive conduit function reflects passive ventricular filling during early diastole (LA/RA Ee) followed by the active emptying booster pump phase (LA/RA Ea) [29]. Strain rate (SR) is defined as the first derivative of the time-strain-curve [30], and can be described as the “speed” of deformation (unit %/s). Briefly, endocardial borders, except for LV analyses for which endo- and epicardial borders are considered, were contoured by the observer in end-diastole and an algorithm was applied to identify the endocardial border in all frames. All generated contours were reviewed and in case of errors, only the manually contoured end-diastolic borders were adjusted and the algorithm executed again. LV and LA functions were analysed in 2-CV and 4-CV views for longitudinal strain and SR. As per recommendations for echocardiography [31] and common practice in CMR analyses [32, 33], pulmonary veins and the left atrial appendage were not included in the delineation of the left atrial endocardial circumference. LV function was also assessed in SA slices for GCS and SR assessment. Papillary muscles were included as part of the blood pool volume, as per general recommendations [34]. RV and RA strains were measured in 4-CV orientations only. Peak ventricular strain and SR values were taken from the strain (rate) curve plotted for the entire cardiac cycle. Additionally, atrial strain and SR values were extracted for specific atrial functional phases including Es, Ee and Ea strains [26]. Automated strain curves were visually reviewed and discarded in case of tracking failure. Final strain values were based on the average of three independent measurements by the same observer to decrease variability. End-diastolic LV mass, LV/RV end-systolic and -diastolic volumes (ESV/EDV) were measured on SAX stacks using commercially available software (QMass^®^ Version 8.1.154.2, Medical Imaging Systems, Leiden, Netherlands). As outlined for the process of CMR-FT, papillary muscles were included as part of the blood pool volume for volume quantifications [34].

### Statistical analyses

The pigs were divided in four groups: (1) baseline pre-intervention PS, (2) baseline pre-intervention PR, (3) post- intervention pigs with PS and (4) post-intervention pigs with PR. Continuous variables are given as median values and interquartile range. The Wilcoxon signed-rank test was used for the comparison between pre-intervention and the respective post-intervention groups (PS and PR), and the non-parametric Mann-Whitney-U test was used for comparison between the two pre-intervention groups and between the two post-intervention groups (PS vs. PR).

Statistical calculations were performed using IBM SPSS Statistic Software Version 26 for Windows (IBM, Armonk, NY, USA) and Microsoft Excel Version 16.72 for Mac. Effects were considered significant for a two-tailed significance level of 5% (p-value below 0.05).

## Results

Clinical assessment showed no signs of heart failure, as previously reported [25].

### PS/PR baseline comparison

Pre-intervention comparisons between pigs who would later receive either PS or PR surgery are reported in Table 1. The median values of several parameters differed, namely RV GLS, right ventricular ejection fraction (RVEF), LA Es, LA Ee, RA Es and RA Ee), however not reaching statistical significance (*p* > 0.05). In general, there were no statistically significant differences between both groups including heart rate, volumetric and deformation based assessments.

**Table 1:** Pre-intervention strain, strain rate and volumetric parameters. The table reports CMR parameters for the subgroup of pigs before pulmonary stenosis (PS) and pulmonary regurgitation (PR) intervention respectively. Data are reported as median values with interquartile range (IQR). Comparisons were made between the pre-PS and pre-PR groups using the nonparametric Mann-Whitney-U-Test. Statistically significant (*p* < 0.05) numbers are given in bold. Abbreviations: GLS: global longitudinal strain, GCS: global circumferential strain, SR: strain rate, EDV: enddiastolic volume, ESV: endsystolic volume, SV: stroke volume, EF: ejection fraction. L/RA: Left/right atrium, Es: Reservoir strain, Ee: conduit strain, Ea: Booster pump strain

Variable	Pre-PS Median (IQR) (*n* = 7)	Pre-PR Median (IQR) (*n* = 7)	*p*
Heart rate	100 (93; 106)	105 (101; 115)	0.165
**Left ventricle**			
GLS (%)	-10.89 (-14,51; -9.63)	-12.43 (-15.58; -11.54)	0.383
GLS SR (%/s)	-0.71 (-0.95; -0.51)	-0.83 (-0.89; -0.71)	0.383
GCS (%)	-19.49 (-20.48; -15.70)	-19.58 (-23.77; -15.80)	1.000
GCS SR (%/s)	-1.33 (-1.53; -1.18)	-1.15 (-1.68; -1.01)	0.620
Mass (g)	32.51 (32.21; 40.57)	39.54 (33.69; 44.87)	0.209
EDV (ml)	72.07 (65.28; 74.78)	71.55 (60.12; 74.99)	0.902
ESV (ml)	46.83 (38.83; 58.83)	48.22 (37.46; 49.18)	1.000
SV (ml)	23.70 (15.95; 26.16)	23.33 (20.07; 25.82)	0.710
EF (%)	35.84 (21.32; 36.50)	34.43 (32.61; 38.37)	1.000
**Right ventricle**			
GLS (%)	-11.73 (-17.99; -9.85)	-20.50 (-22.65; -15.49)	0.128
GLS SR (%/s)	-0.82 (-1.11; -0.79)	-1.25 (-1.30; -0.99)	0.097
EDV (ml)	60.67 (44.94; 63.67)	58.11 (55.72; 72.25)	0.318
ESV (ml)	33.38 (28.12; 44.24)	32.27 (26.40; 49.06)	0.902
SV (ml)	20.35 (9.82; 30.39)	27.38 (18.74; 29.70)	0.383
EF (%)	31.50 (16.95; 53.65)	49.57 (32.09; 52.15)	0.456
**Left atrium**			
LA-Es (%)	26.00 (19.98; 81.70)	40.48 (36.51; 51.48)	0.620
LA-Es SR (%/s)	1.78 (1.26; 2.41)	1.47 (1.34; 1.80)	0.535
LA-Ee (%)	23.78 (13.52; 68.25)	35.44 (25.58; 39.94)	0.710
LA-Ee SR (%/s)	-1.34 (-1.80; -0.63)	-1.40 (-1.97; -1.01)	0.456
LA-Ea (%)	6.46 (5.15; 13.45)	8.14 (0.76; 22.64)	0.902
LA-Ea SR (%/s)	-1.44 (-3,95; -1.13)	-1.67 (-1.97; -1,21)	0.805
**Right atrium**			
RA-Es (%)	23.20 (18.99; 32.47)	31.65 (29.90; 52.58)	0.073
RA-Es SR (%/s)	1.07 (0.79; 1.17)	1.71 (1.25; 2.04)	0.165
RA-Ee (%)	15.04 (10.96; 28.64)	20.63 (16.05; 24.19)	0.805
RA-Ee SR (%/s)	-0.83 (-1.17; -0.72)	-1.39 (-1.55; -0.61)	0.710
RA-Ea (%)	6.14 (0.00; 10.82)	7.76 (0.00; 18.11)	0.710
RA-Ea SR (%/s)	-0.85 (-1.43; -0.73)	-1.84 (-2.23; -0.57)	0.318

### Impact of PS and PR intervention on cardiac function

**Fig. 2:**
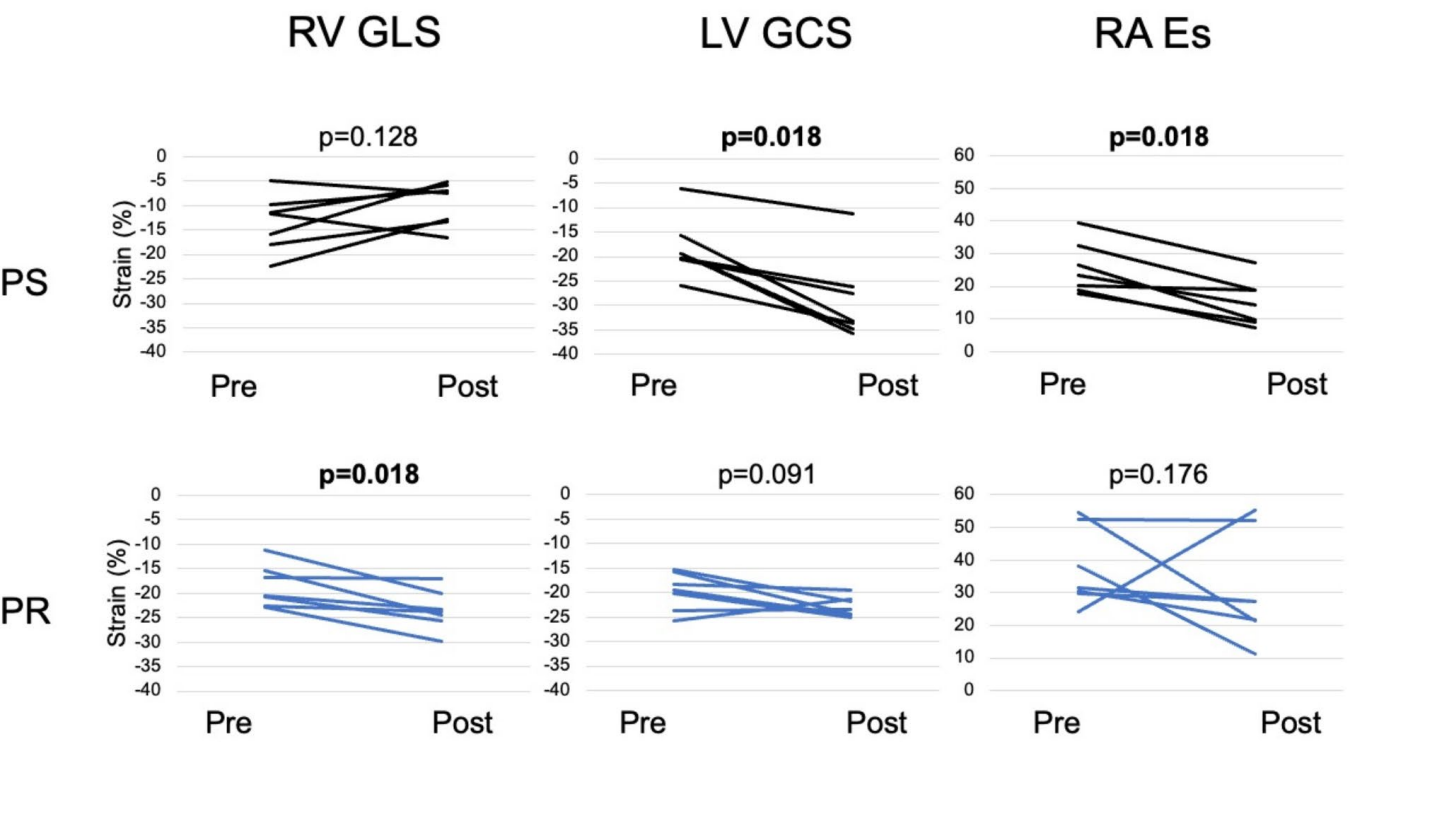
Line charts for deformation parameters pre and post intervention in PS and PR This table reports changes over time between pre- and post-intervention (after 10–12 weeks) in PS (*n* = 7, upper row) and PR pigs (*n* = 7, lower row) for RV GLS (left column), LV GCS (middle column) and RA Es (right column). Y-Axis: Strain in %. X-Axis: Time. Abbreviations: RV: Right ventricle, GLS: Global longitudinal strain, LV: Left ventricle, GCS: Global circumferential strain, RA: Right atrium, Es: Reservoir strain, PS: Pulmonary stenosis, PR: Pulmonary regurgitation, CMR-FT: Cardiac magnetic resonance feature tracking

Data on the comparison of pigs before and after PS is shown in Table 2; Fig. 2. Heart rate at the time of the respective CMR scans was significantly lower in pigs after PS intervention (before 100 bpm vs. after 79 bpm, *p* = 0.028).

**Table 2:** Strain, strain rate and volumetric parameters pre- and post-PS-intervention. The table reports CMR parameters for the subgroup of pigs before pulmonary stenosis (PS) and after PS model creation. Data are reported as median values with interquartile range (IQR). Comparisons were made between the pre-PS and post-PS groups using the Wilcoxon-signed-rank-test. Statistically significant (*p* < 0.05) numbers are given in bold. Abbreviations: GLS: global longitudinal strain, GCS: global circumferential strain, SR: strain rate, EDV: enddiastolic volume, ESV: endsystolic volume, SV: stroke volume, EF: ejection fraction. L/RA: Left/right atrium, Es: Reservoir strain, Ee: conduit strain, Ea: Booster pump strain

Variable	Pre-PS Median (IQR) (*n* = 7)	Post-PS Median (IQR) (*n* = 7)	*p*
Heart rate	100 (93; 106)	79 (69; 83)	**0.028**
**Left ventricle**			
GLS (%)	-10.89 (-14,51; -9.63)	-13.62 (-16.60; -9.89)	0.499
GLS SR (%/s)	-0.71 (-0.95; -0.51)	-0.75 (-0.77; -0.55)	0.310
GCS (%)	-19.49 (-20.48; -15.70)	-33.21 (-34.76; -26.20)	**0.018**
GCS SR (%/s)	-1.33 (-1.53; -1.18)	-1.81 (-1.99; -1.29)	0.063
Mass (g)	32.51 (32.21; 40.57)	79.66 (70.73; 86.92)	**0.018**
EDV (ml)	72.07 (65.28; 74.78)	92.04 (80.94; 109.27)	**0.018**
ESV (ml)	46.83 (38.83; 58.83)	46.29 (6.00; 56.00)	0.398
SV (ml)	23.70 (15.95; 26.16)	51.35; (38.85; 54.85)	**0.018**
EF (%)	35.84 (21.32; 36.50)	55.71 (42.21; 60.76)	**0.018**
**Right ventricle**			
GLS (%)	-11.73 (-17.99; -9.85)	-7.51 (-13.25; -5.97)	0.128
GLS SR (%/s)	-0.82 (-1.11; -0.79)	-0.57 (-0.67; -0.34)	**0.018**
EDV (ml)	60.67 (44.94; 63.67)	118.09 (94.74; 141.56)	**0.018**
ESV (ml)	33.38 (28.12; 44.24)	77.88 (53.14; 82.74)	**0.018**
SV (ml)	20.35 (9.82; 30.39)	41.80 (35.35; 55.26)	**0.018**
EF (%)	31.50 (16.95; 53.65)	38.98 (29.93; 43.90)	0.735
**Left atrium**			
LA-Es (%)	26.00 (19.98; 81.70)	36.59 (17.46; 38,36)	0.091
LA-Es SR (%/s)	1.78 (1.26; 2.41)	1.56 (1.02; 2.34)	0.176
LA-Ee (%)	23.78 (13.52; 68.25)	23.18 (15.10; 24.88)	0.176
LA-Ee SR (%/s)	-1.34 (-1.80; -0.63)	-1.67 (-2.00; -1.22)	0.398
LA-Ea (%)	6.46 (5.15; 13.45)	9.64 (0.92; 13.49)	0.735
LA-Ea SR (%/s)	-1.44 (-3,95; -1.13)	-0.82 (-1.07; -0.16)	0.018
**Right atrium**			
RA-Es (%)	23.20 (18.99; 32.47)	14.22 (8.97; 18.93)	**0.018**
RA-Es SR (%/s)	1.07 (0.79; 1.17)	0.61 (0.53; 0.99)	**0.018**
RA-Ee (%)	15.04 (10.96; 28.64)	8.65 (5.59; 12.42)	**0.028**
RA-Ee SR (%/s)	-0.83 (-1.17; -0.72)	-0.35 (-0.60; -0.30)	**0.043**
RA-Ea (%)	6.14 (0.00; 10.82)	4.26 (1.80; 10.28)	0.735
RA-Ea SR (%/s)	-0.85 (-1.43; -0.73)	-0.47 (-0.65; -0.42)	0.128

#### Right cardiac function

Volumetric assessments of RV morphology and function showed RV dilatation. RV EDV and ESV were increased following PS procedure (*p* = 0.018 for both). This resulted in increased SV (*p* = 0.018) with changes in RVEF not reaching statistical significance (*p* = 0.735). RV contractility decreased following PS procedure as shown by a significant decrease in RV GLS SR (before − 0.82 vs. after − 0.57, *p* = 0.018) paralleled by a numerical but statistically non-significant decrease in RV GLS (before − 11.73 vs. after − 7.51, *p* = 0.128). In line, RA function deteriorated. Both RA Es (*p* = 0.018) and associated SR (*p* = 0.018) as well as Ee (*p* = 0.028) and associated SR (*p* = 0.043) decreased significantly.

#### Left cardiac function

In contrast, left cardiac function improved following PS induction. This could be demonstrated by increased LV stroke volume (SV) and left ventricular ejection fraction (LVEF) based on increased EDV (*p* = 0.018 for all) alongside non-significantly changed ESV (*p* = 0.398). Furthermore, LV contractility increased as appreciated from GCS (before − 19.49 vs. after − 33.21, *p* = 0.018). GLS did not change significantly (before − 10.89 vs. after − 13.62, *p* = 0.499).

Data for the comparison of pigs before and after PR is given in Table 3; Fig. 2.

**Table 3:** Strain, strain rate and volumetric parameters pre- and post-PR-intervention. The table reports CMR parameters for the subgroup of pigs before pulmonary regurgitation (PR) and after PR model creation. Data are reported as median values with interquartile range (IQR). Comparisons were made between the pre-PR and post-PR groups using the Wilcoxon-signed-rank-test. Statistically significant (*p* < 0.05) numbers are given in bold. Abbreviations: GLS: global longitudinal strain, GCS: global circumferential strain, SR: strain rate, EDV: enddiastolic volume, ESV: endsystolic volume, SV: stroke volume, EF: ejection fraction. L/RA: Left/right atrium, Es: Reservoir strain, Ee: conduit strain, Ea: Booster pump strain

Variable	Pre-PR median (IQR) (*n* = 7)	Post-PR median (IQR) (*n* = 7)	*p*
Heart rate	105 (101; 115)	96 (90; 107)	0.236
**Left ventricle**			
GLS (%)	-12.43 (-15.58; -11.54)	-15.18 (-15.81; -12.92)	0.398
GLS SR (%/s)	-0.83 (-0.89; -0.71)	-0.82 (-0.94; -0.77)	0.735
GCS (%)	-19.58 (-23.77; -15.80)	-23.50 (-24.50; -21.29)	0.091
GCS SR (%/s)	-1.15 (-1.68; -1.01)	-1.51 (-1.67; -1.19)	0.237
Mass (g)	39.54 (33.69; 44.87)	74.76 (61.41: 87.70)	**0.018**
EDV (ml)	71.55 (60.12; 74.99)	95.46 (87.04; 108.59)	**0.018**
ESV (ml)	48.22 (37.46; 49.18)	49.77 (46.29; 55.64)	0.237
SV (ml)	23.33 (20.07; 25.82)	48.13 (44.57; 55.71)	**0.018**
EF (%)	34.43 (32.61; 38.37)	51.18 (41.05; 52.58)	**0.018**
**Right ventricle**			
GLS (%)	-20.50 (-22.65; -15.49)	-23.84 (-25.61; -20.09)	**0.018**
GLS SR (%/s)	-1.25 (-1.30; -0.99)	-1.24 (-1.27; -1.16)	0.499
EDV (ml)	58.11 (55.72; 72.25)	149.69 (103.95; 181.80)	**0.018**
ESV (ml)	32.27 (26.40; 49.06)	80.76 (61.29; 92.87)	**0.018**
SV (ml)	27.38 (18.74; 29.70)	69.06 (42.66; 92.64)	**0.018**
EF (%)	49.57 (32.09; 52.15)	46.02 (41.36; 50.96)	0.398
**Left atrium**			
LA-Es (%)	40.48 (36.51; 51.48)	44.32 (30.80; 45.13)	0.612
LA-Es SR (%/s)	1.47 (1.34; 1.80)	1.31 (1.19; 1.49)	0.128
LA-Ee (%)	35.44 (25.58; 39.94)	31.66 (21.21; 45.13)	1.000
LA-Ee SR (%/s)	-1.40 (-1.97; -1.01)	-1.80 (-2.44; -1.24)	0.398
LA-Ea (%)	8.14 (0.76; 22.64)	8.73 (0.00; 19.04)	0.463
LA-Ea SR (%/s)	-1.67 (-1.97; -1,21)	-1.23 (-1.51; -0.05)	**0.043**
**Right atrium**			
RA-Es (%)	31.65 (29.90; 52.58)	27.34 (21.21; 52.41)	0.176
RA-Es SR (%/s)	1.71 (1.25; 2.04)	0.86 (0.79; 1.72)	0.128
RA-Ee (%)	20.63 (16.05; 24.19)	20.51 (15.75; 43.86)	0.499
RA-Ee SR (%/s)	-1.39 (-1.55; -0.61)	-1.10 (-1.58; -0.57)	0.866
RA-Ea (%)	7.76 (0.00; 18.11)	2.26 (0.00; 6,89)	0.249
RA-Ea SR (%/s)	-1.84 (-2.23; -0.57)	-0.83 (-1.20; -0.12)	**0.046**

Heart rate at the time of the respective CMR scans did not differ significantly after PR intervention (before 105 bpm vs. after 96 bpm, *p* = 0.236).

#### Right cardiac function

Similar to PS, following PR surgery, RV EDV, ESV and SV were significantly increased (*p* = 0.018) with changes in RVEF remaining statistically non-significant (*p* = 0.398). In contrast to PS however, RV contractility increased as appreciated from an increase in RV GLS (before − 20.50 vs. after − 23.84, *p* = 0.018). In line and opposed to PS, there was no statistically significant decrease of RA Es (*p* = 0.176).

#### Left cardiac function

Similarly to PS, LV EDV (*p* = 0.018) but not ESV (*p* = 0.237) increased significantly resulting in increased SV and LVEF (*p* = 0.018 for both). However, as opposed to PS, there was no statistically significant increase in LV strain and SR following PR surgery (*p* ≥ 0.091).

### PS/PR follow-up comparison

Comparisons between the postintervention groups (PS and PR) are reported in Table 4. Heart rate at the time of the respective CMR scans was significantly lower in pigs with PS intervention compared to PR pigs (PR 96 bpm vs. PS 79 bpm, *p* = 0.007).

**Table 4:** Post-intervention strain, strain rate and volumetric parameters. The table reports CMR parameters for the subgroup of pigs after pulmonary stenosis (PS) and after pulmonary regurgitation (PR) intervention respectively. Data are reported as median values with interquartile range (IQR). Comparisons were made between the post-PS and post-PR groups using the nonparametric Mann-Whitney-U-Test. Statistically significant (*p* < 0.05) numbers are given in bold. Abbreviations: GLS: global longitudinal strain, GCS: global circumferential strain, SR: strain rate, EDV: enddiastolic volume, ESV: endsystolic volume, SV: stroke volume, EF: ejection fraction. L/RA: Left/right atrium, Es: Reservoir strain, Ee: conduit strain, Ea: Booster pump strain

Variable	Post PS Median (IQR) (*n* = 7)	Post PR Median (IQR) (*n* = 7)	*p*
Heart rate	79 (69; 83)	96 (90; 107)	**0.007**
**Left ventricle**			
GLS	-13.62 (-16.60; -9.89)	-15.18 (-15.81; -12.92)	0.805
GLS SR	-0.75 (-0.77; -0.55)	-0.82 (-0.94; -0.77)	**0.038**
GCS	-33.21 (-34.76; -26.20)	-23.50 (-24.50; -21.29)	**0.026**
GCS SR	-1.81 (-1.99; -1.29)	-1.51 (-1.67; -1.19)	0.456
Mass	79.66 (70.73; 86.92)	74.76 (61.41: 87.70)	0.535
EDV	92.04 (80.94; 109.27)	95.46 (87.04; 108.59)	0.710
ESV	46.29 (6.00; 56.00)	49.77 (46.29; 55.64)	0.318
SV	51.35; (38.85; 54.85)	48.13 (44.57; 55.71)	0.805
EF	55.71 (42.21; 60.76)	51.18 (41.05; 52.58)	0.383
**Right ventricle**			
GLS	-7.51 (-13.25; -5.97)	-23.84 (-25.61; -20.09)	**< 0.001**
GLS SR	-0.57 (-0.67; -0.34)	-1.24 (-1.27; -1.16)	**< 0.001**
EDV	118.09 (94.74; 141.56)	149.69 (103.95; 181.80)	0.259
ESV	77.88 (53.14; 82.74)	80.76 (61.29; 92.87)	0.259
SV	41.80 (35.35; 55.26)	69.06 (42.66; 92.64)	**0.038**
EF	38.98 (29.93; 43.90)	46.02 (41.36; 50.96)	**0.038**
**Left atrium**			
LA-Es	36.59 (17.46; 38,36)	44.32 (30.80; 45.13)	0.053
LA-Es SR	1.56 (1.02; 2.34)	1.31 (1.19; 1.49)	0.383
LA-Ee	23.18 (15.10; 24.88)	31.66 (21.21; 45.13)	**0.038**
La-Ee SR	-1.67 (-2.00; -1.22)	-1.80 (-2.44; -1.24)	0.318
LA-Ea	9.64 (0.92; 13.49)	8.73 (0.00; 19.04)	1.000
LA-Ea SR	-0.82 (-1.07; -0.16)	-1.23 (-1.51; -0.05)	0.318
**Right atrium**			
RA-Es	14.22 (8.97; 18.93)	27.34 (21.21; 52.41)	**0.017**
RA-Es SR	0.61 (0.53; 0.99)	0.86 (0.79; 1.72)	0.209
RA-Ee	8.65 (5.59; 12.42)	20.51 (15.75; 43.86)	**0.004**
RA-Ee SR	-0.35 (-0.60; -0.30)	-1.10 (-1.58; -0.57)	**0.011**
RA-Ea	4.26 (1.80; 10.28)	2.26 (0.00; 6,89)	0.383
RA-Ea SR	-0.47 (-0.65; -0.42)	-0.83 (-1.20; -0.12)	0.209

#### Right cardiac function

Right cardiac function was significantly better following PR compared to PS surgery.

This included RV volumetric SV and RVEF (*p* = 0.038 for both) and deformation based assessments RV GLS (PR -23.84 vs. PS -7.51, *p* < 0.001) as well as associated SR (PR -1.24 vs. PS -0.57, *p* < 0.001). In line RA function was higher in PR compared to PS including RA Es (PR 27.34 vs. PS 14.22, *p* = 0.017) and Ee (PR 20.51 vs. PS 8.65, *p* = 0.004).

#### Left cardiac function

In contrast, LV function contractility improved in PS compared to PR as appreciated from LV GCS (PR -23.50 vs. PS -33.21, *p* = 0.026) whilst overall volumes did not differ significantly (*p* ≥ 0.318).

Results of multivariable linear regressions to predict differences (post-intervention minus pre-intervention values) from two variables (type of intervention, respective pre-intervention values) are reported in Table 5. Parameters from Table 4 with *p* < 0.1 were included. For the prediction of heart rate, RVSV, RA Es and RA SR, the type of intervention did not reach statistical significance after controlling for baseline values. The post-pre differences for all the other parameters were confirmed to be significantly associated to type of intervention after controlling for baseline values. Additionally, LA Es, not reaching statistical significance in Table 4, showed a significant association with type of intervention in this analysis after controlling for baseline values.

**Table 5:** Linear regression of strain, strain rate and volumetric parameters. The table reports results of linear regression models using CMR parameters for pigs after pulmonary stenosis (PS) and after pulmonary regurgitation (PR) intervention, as a whole. Linear regression was performed using two independent variables: first, type of intervention (PS vs. PR); second, pre-intervention value of any given parameter. The dependent variable was the difference between post-intervention and pre-intervention values. All parameters from Table 4 with *p* < 0.1 were included in the analysis. Data are reported as unstandardized b coefficients with respective p-values. Statistically significant (*p* < 0.05) numbers are given in bold. Abbreviations: GLS: Global longitudinal strain, SR: Strain rate, GCS: Global circumferential strain, SV: Stroke volume, EF: Ejection fraction. LA/RA: Left/right atrium, Es: Reservoir strain, Ee: Conduit strain

Variable (difference post/pre-intervention)	Unstandardized B for PS vs. PR	*P* for PS vs. PR	Unstandardized B for baselines	*P* for PS vs. PR baseline
Heart rate	0.338	0.972	0.015	0.977
**Left ventricle**				
GLS SR	-0.17	**0.039**	-0.79	**0.001**
GCS	7.33	**0.022**	-0.23	0.427
**Right ventricle**				
GLS	-11.65	**< 0.001**	-0.60	**0.020**
GLS SR	-0.56	**< 0.001**	-0.52	**0.004**
SV	27.27	0.173	-0.01	0.993
EF	8.27	**0.035**	-0.98	**< 0.001**
**Left atrium**				
LA-Es	14.60	**0.019**	-0.72	**< 0.001**
LA-Ee	13.55	**0.014**	-0.88	**< 0.001**
**Right atrium**				
RA-Es	14.07	0.121	-0.84	**0.048**
RA-Ee	18.20	**0.019**	-0.93	0.063
RA-Ee SR	-0.89	0.051	-0.56	0.099

## Discussion

The present study reports the differing impact of PS/PR induction in a porcine model based on state-of-the-art CMR imaging. PS but not PR resulted in deteriorated right cardiac function. First, whilst both increased afterload (PS) and preload (PR) resulted in increased RV volumes, RV deformation deteriorated following prompt increase in afterload caused by PS but improved following increased preload caused by PR. Secondly, volume overload and congestion following RV functional deterioration resulted in consecutive RA functional impairment in PS but not PR. Lastly, LV deformation significantly improved in PS but not PR potentially compensating for impaired LV preload to maintain LV SV.

### Differing effect of pre- and afterload on RV deformation parameters

Myocardial deformation as appreciated from strain is load dependent. As demonstrated by the Frank-Starling mechanism for the LV, both increased preload and afterload lead to volume overload and subsequently increased deformation. Indeed, both PS and PR resulted in increased RV dilatation with subsequently higher SV. However, there were significant differences for PS and PR in RV strain. An increase in RV afterload in PS has been related to deteriorated RV strains, at least on a segmental level [35]. This has also been demonstrated in a population of HFpEF patients, where patients with raised pulmonary vascular resistance exhibited impaired longitudinal RV strain [36]. In line, we demonstrated significantly decreased RV GLS SR and a numerical but not significant decrease of overall RV GLS. However, increased RV SV has previously been attributed to increased RV end-systolic elastance in PS [24]. One the one hand side this seemingly contraintuitive finding may thus correspond to an increase in circumferential/radial RV contractility. On the other hand side overall dilatation with increased SV but not EF may merely represent limited forms of compensation and not overall improved contractility. Intriguingly, sole increase in preload in PR without the aspect of additionally increased afterload from PS led to a statistically significant increase in RV GLS. This can either be interpreted as (a) a consequence of the Frank-Starling mechanism or (b) as an increase in intrinsic contractility. Of note, RV GLS SR was largely unchanged in PR pigs compared to pre-intervention. Strain rate is generally considered to be a marker which more closely reflects contractile function and is less load-dependent than strain [37]. On balance, this suggests the Frank-Starling mechanism as the driving force behind the effect, as was discussed for mitral and aortic regurgitation previously [38]. Nonetheless, these results underline that the RV can handle volume overload better than pressure overload, at least in the timeframe studied here.

Development of PS increases RV pressure in a chronic manner, resulting in RV morphological and structural adaptations [39]. The effect of increased pressure load on myocardial tissue is a double-edged sword. On the one hand side concentric hypertrophy helps to overcome increased afterload, on the other hand side tissue remodelling includes fibrosis with subsequently reduced ventricular compliance [40]. Initially, as shown for the left ventricle, concentric hypertrophy enables the ventricle to maintain wall stress at lower levels throughout the cardiac cycle despite increases in pressure, and therefore was historically regarded as favourable adaptation [7, 41]. However, maladaptation to the increased afterload may ensue in the form of eccentric hypertrophy or dilatation, resulting in ventricular failure [40], calling into question the positive view of concentric hypertrophy. Adding to this, adverse histological and functional findings as well as reduced survival rates were noted in a murine model of pressure compared to volume overload [9]. In addition, an abruptly and severely increased pressure load can cause RV decompensation with life-threatening consequences. This can be the case with acute pulmonary embolism since the RV is not able to respond to the quick rise in afterload with remodelling [42], which might be best suited for comparison with acute induction of PS by surgery.

Unlike mild volume overload, which can be handled by dynamic modulation of SV [43], continuous severe volume overload may induce myocardial remodelling with subsequent heart failure [44]. In contrast to afterload-induced remodelling with thickening of the ventricle, preload mainly results in a dilated, thin-walled ventricle [45, 46]. However, the literature for the LV suggests that these effects only develop over time, with a proposed timeframe of up to 21 weeks of severe volume overload until systolic failure occurs [45]. Despite this potential danger of heart failure in volume overload in the long run, our study showed that at least in a timeframe of 10 to 12 weeks, the RV can better cope with volume overload than with pressure overload. This is in line with earlier findings based on four dimensional phase contrast magnetic resonance [25].

### Right atrial involvement in PS and PR

Over the course of RV hypertrophy development, RV compliance may diminish, translating to hindered diastolic function, as previously shown in clinical and experimental settings of chronic RV pressure overload [47, 48]. Accordingly, we observed a statistically significant decrease of total RA strain, paralleled by a reduction in passive RA conduit strain of almost the same magnitude in the PS model. As conduit strain reflects passive ventricular filling [49], this finding is indicative of increased RV stiffness as an adverse by-product of hypertrophy [48]. Furthermore, RA functional failure in PS may indicate overall congestion introduced by RV functional failure in PS as opposed to PR. In PR, a previous study found an increased RV compliance based on invasive pressure-volume loops [24], translating to improved RV diastolic function. In the present PR population, there was no distinct impact on RA physiology except for a significant reduction for RA Ea strain rate and a numerical statistically non-significant trend for a decline in active RA Ea. This could imply that their RV filling is less dependent on active atrial contraction due to improved passive filling. Altogether, these RA results are in line with our findings for the RV, as they indicate significant right cardiac failure in PS corresponding to a more maladaptive response to pressure than volume overload.

### LV compensation of RV dysfunction

Pigs from both PR and PS interventions showed increased LV mass and EDV. With ESV remaining similar, this resulted in increased SV and EF. However, only pigs following PS induction displayed a significant increase in LV GCS compared to pre-intervention as well as post-intervention PR pigs. First, this may suggest a compensatory effect of the LV to uphold cardiac output despite reduced preload caused by RV functional failure in PS. Second, this hint at circumferential compensation echoes findings from patients with pulmonary hypertension: in spite of diminished longitudinal LV function, their overall systolic LV function stayed the same, owing to an increase in lateral movement [50]. From a mechanistic standpoint, the LV and RV are intricately linked on several layers, such as their shared septum and common myocardial fibres. This allows for functional interplay [51–54]. Estimates for contribution of LV contraction to RV systolic function range as high as > 50% [54]. Consequently, improved LV contractility as appreciated from increased LV GCS may explain the previously reported increases of RV end-systolic elastance as an indication of improved contractility in PS [24], despite a tendency for RV GLS to deteriorate. Accordingly, in the previously published analysis of pigs studied here, LV Ees was numerically increased in PS, albeit not reaching statistical significance [24].

### Advantages of the employed animal model

Using large animal models helps to close the gap between small animal laboratory studies and clinical trials. In a cellular and mouse model adverse effects of afterload as opposed to preload on left ventricular remodelling and function have been demonstrated [9]. These findings are expanded to RV physiology incorporating a model more closely resembling human heart physiology [56]. In addition, the advantages of using large animal models for cardiovascular disease exploration lies in their body size allowing for easier surgical manipulation and more detailed pathophysiological assessment in the setting of imaging studies [57]. The utilized animal model also allowed for the induction of specific changes in pre- and afterload, respectively, which enabled us to observe alterations of myocardial deformation over time attributed to the artificially created hemodynamic differences. During the original experiments, echocardiographic control was established during the process of model creation to achieve maximum pulmonary artery stenosis possible [25] Presence of pulmonary regurgitation was also confirmed using echo. Therefore, loading conditions (increase of pre-/afterload) in the respective groups at time of model creation were experimentally ensured.

Furthermore, the use of CMR-FT for deformation analysis in landrace pigs has already been demonstrated to show low intra- and interobserver variability for the assessment of LV GLS and GCS [58]. CMR FT technology is already available for patient care and needs no specific transfer from the animal model to the human.

### Outlook for further research and possible clinical applications

Our findings underline the potential utility of CMR-FT in a porcine model to track changes caused by PS and PR induction. Based on this, the model might be used in the future to assess the long-term effect of PS vs. PR as well as targeted therapeutic interventions respectively, as part of a longitudinal study. In line, assessment of the clinical outcome within the porcine model would be of interest to evaluate the possible prognostic implications.

Our results show the capability of deformation analyses to detect changes in all four chambers in PS and PR models. Indeed, current imaging guidelines relating to repaired TOF patients hint at the option of ventricular assessment via deformation analysis, whilst to date volumetric analyses remain the reference standard [59]. Based on our results, the assessment of deformation imaging should be evaluated further in the future, as they offer additional pathophysiological information over established imaging parameters. If proven useful in valvular disease, it could facilitate early detection of ventricular dysfunction to monitor patients and potentially guide early treatment decisions. Indeed, strain analysis is already a relevant research focus in the realm of aortic stenosis. For instance, published literature shows the value of CMR-FT for identification of LV dysfunction, risk stratification and assessment of treatment effect after transcatheter valve implantation [60].

### Limitations

Our analysis offers insights into the pathophysiology of volume vs. pressure overload in the right ventricle. Due to this study’s nature as a subanalysis, its limitations overlap with those of previous analyses based on the same study [24, 25]: The small population of pigs likely renders it underpowered for the identification of subtle, within and between-group differences. However, the fact that in several key parameters, significant differences were detected despite the small sample size, underscores the robustness of CMR-FT to pick up change in myocardial function. Also, as the two CMR scans were conducted at the relatively fixed interval of 10 to 12 weeks, any acute or subacute changes in between or long-term effects potentially occurring after the study could not be assessed using this design. Additionally, due to the primary study design, clinical outcome of pigs was not recorded in detail [25].

Furthermore, as can be appreciated from Table 1, there were striking differences in median values in several key parameters before surgical interventions, although these differences did not reach statistical significance. As shown in Table 5, after accounting for these non-significant differences in baseline values, there was still a significant association of the type of intervention (PS/PR) with most of the parameters studied. This underlines the strong effect of the surgical procedure on clinical and imaging parameters studied.

The most obvious clinical example to which our animal study might be applied is TOF patients before (PS group) and after repair, once regurgitation has set in (PR group). This translation however is somewhat limited, as repaired TOF patients experience the cumulative effect of already present pressure overload (from their baseline disease) and volume overload (from newly developed pulmonary regurgitation). In contrast, in our study, the pigs undergoing artificial creation of PR were healthy before the intervention. As such, a previously published experimental design focused on the induction of PR in an already pressure-overloaded RV [61].

Lastly, circumferential or radial motion might compensate for EF in RV physiology in case of impaired longitudinal contraction. However, we did neither analyze GCS nor GRS for the RV and as such cannot assess this potential compensation mechanism.

## Conclusion

In a porcine model of surgically created PS and PR, CMR-FT was able to detect cardiac functional differences following intervention. Whilst PS resulted in overall right cardiac functional deterioration, PR did not. This highlights differing effects of pre- and afterload on RV physiology – which is more prone to acute changes in afterload as opposed to the LV.

## Data Availability

No datasets were generated or analysed during the current study.
